# Senescence-associated-β-galactosidase staining following traumatic brain injury in the mouse cerebrum

**DOI:** 10.1371/journal.pone.0213673

**Published:** 2019-03-11

**Authors:** Tadasuke Tominaga, Ryo Shimada, Yoshikazu Okada, Takakazu Kawamata, Kazuhiko Kibayashi

**Affiliations:** 1 Department of Neurosurgery, School of Medicine, Tokyo Women’s Medical University, Tokyo, Japan; 2 Department of Legal Medicine, School of Medicine, Tokyo Women’s Medical University, Tokyo, Japan; 3 Department of Neurosurgery, St. Luke’s International Hospital, Tokyo, Japan; University of Modena and Reggio Emilia, ITALY

## Abstract

Primary and secondary traumatic brain injury (TBI) can cause tissue damage by inducing cell death pathways including apoptosis, necroptosis, and autophagy. However, similar pathways can also lead to senescence. Senescent cells secrete senescence-associated secretory phenotype proteins following persistent DNA damage response signaling, leading to cell disorders. TBI initially activates the cell cycle followed by the subsequent triggering of senescence. This study aims to clarify how the mRNA and protein expression of different markers of cell cycle and senescence are modulated and switched over time after TBI. We performed senescence-associated-β-galactosidase (SA-β-gal) staining, immunohistochemical analysis, and real-time PCR to examine the time-dependent changes in expression levels of proteins and mRNA, related to cell cycle and cellular senescence markers, in the cerebrum during the initial 14 days after TBI using a mouse model of controlled cortical impact (CCI). Within the area adjacent to the cerebral contusion after TBI, the protein and/or mRNA expression levels of cell cycle markers were increased significantly until 4 days after injury and senescence markers were significantly increased at 4, 7, and 14 days after injury. Our findings suggested that TBI initially activated the cell cycle in neurons, astrocytes, and microglia within the area adjacent to the hemicerebrum contusion in TBI, whereas after 4 days, such cells could undergo senescence in a cell-type-dependent manner.

## Introduction

Primary and secondary traumatic brain injury (TBI) can cause tissue damage by inducing cell death pathways including apoptosis, necroptosis, and autophagy [1]. However, similar pathways can also lead to senescence [2, 3].

Historically, neurons, as differentiated post-mitotic cells, were considered incapable of re-entering the cell cycle and were thought to remain permanently in the G0 phase. However, it is now recognized that mature differentiated post-mitotic neurons also exhibit cell cycle re-entry, inducing apoptosis or neuronal proliferation [4, 5]. Notably, primary and secondary TBI increase the expression of cell cycle-related proteins that are associated with the up-regulation of apoptosis in post-mitotic cells such as neurons. Cell cycle activation also induces the proliferation of astrocytes and microglia in order to activate glial scarring and microglia through the release of inflammatory factors [6].

In particular, cyclin D1 and proliferating cell nuclear antigen (PCNA) represent cell cycle markers activated by TBI [4]. The cyclin D family, which includes cyclin D1–D3, regulates cyclin-dependent kinase (CDK) kinases throughout the cell cycle. Different cyclins exhibit distinct expression and degradation patterns in order to coordinate the timing of each mitotic event. Cyclin D1 forms complexes with regulatory subunits of CDK4 or CDK6, with its activity being required for cell cycle G1/S transition and metastasis. Cyclin D1 interacts with and is positively regulated by the retinoblastoma tumor suppressor protein (RB) [7, 8]. In turn, PCNA, which belongs to the DNA sliding clamp family [9], is a marker of late G1/early S-phase during cell cycle progression [6, 10]. The *PCNA* gene is expressed during cell proliferation and DNA replication processes and plays a pivotal roles in DNA repair pathways, including base excision repair, nucleotide excision repair, and mismatch repair. PCNA positively or negatively controls cell cycle progression and allows sister-chromatid cohesion by interacting with other factors, to prevent inappropriate homologous recombination [9].

In addition to cell cycle arrest and apoptotic cell death, activated cyclin-dependent kinase inhibitor 2A (p16) [11, 12], transformation related protein 53 (p53) [13] and cyclin-dependent kinase inhibitor 1A (p21) [14] induce cellular senescence. In the senescence pathway, p16, p53, and p21 inactivate cyclin D1 [15, 16] and PCNA [17], subsequently inhibiting CDK2, CDK4, and CDK6, then block the G1 to S-phase transition in the cell cycle through activation of the RB family of proteins [17–20].

In turn, senescent cells secrete senescence-associated secretory phenotype proteins, growth factors, proteases, cytokines, and other factors by potent autocrine and paracrine activities following persistent DNA damage response signaling. Senescent cells also can stimulate premalignant and weakly malignant epithelial cells to invade a basement membrane by secreting high levels of interleukin 6 (IL-6) and IL-8 that impact the behavior of neighboring cells, leading to cell disorders [21, 22]. The elevated activity of β-galactosidase (β-gal), a glycoside hydrolase enzyme [23] that serves as a biomarker to identify senescent cells in mammalian tissues, may derive from the increase of some lysosomal enzyme activity in senescent cells [24]. Specifically, at pH 6.0, active β-gal reacts with 5-bromo-4-chloro-3-indoyl-β-d-galactopyranoside (X-gal) [24, 25], a soluble, colorless compound consisting of galactose linked to a substituted indole. Hydrolysis of X-gal by β-gal releases substituted indoles that dimerize spontaneously to give an insoluble strong blue product in tissue or on growth medium [26].

Considering that TBI initially activates the cell cycle whereas these cells may then undergo senescence. In previous studies, TBI induces changes in the expression of β-gal [27], cyclin D1, PCNA [4], p16 [18], p21 [28], and p53 [29]. However, previous studies did not assess whether the expression of different markers of the cell cycle and senescence were modulated over time following TBI. Therefore, this study aims to clarify how TBI changes and switches expression of the above genes over time and whether it is related to senescence. To address this issue, we performed histochemical staining, immunohistochemistry and/or real-time PCR to examine time-dependent changes in these proteins and/or mRNAs in the cerebrum during the first 14 days after TBI, using a controlled cortical impact (CCI) model in mice.

## Materials and methods

### Animals and surgery

The Ethical Review Committee of Animal Experiments at Tokyo Women’s Medical University approved the procedures for the animal experiments (AE18-073). In total, 90 male C57BL/6J mice weighing 22−25 g (about 10 weeks old) were randomly allocated to the control, sham, or injury group. The mice were anesthetized using an intraperitoneal (i.p.) injection of three types of mixed agents: Domitol (medetomidine hydrochloride: 0.3 mg/kg, ZENOAQ, Nippon Zenyaku Kogyo Co. Ltd., Fukushima, Japan), midazolam (4 mg/kg, Fuji Parma Co. Ltd., Toyama, Japan), and butorphanol (5 mg/kg, Meiji Seika Pharma Co. Ltd., Tokyo, Japan) [30], following which 4% isoflurane (Wako Pure Chemical Industries, Co. Ltd.; Osaka, Japan) was delivered using an enclosed chamber and an inhalation anesthesia apparatus (Narcobit-E; Natsume Seisakusho, Tokyo, Japan) [31]. The mice were placed in a stereotaxic frame, and their scalps were shaved and cut open in order to expose the skull. Their rectal temperature was maintained at 37°C using a warming pad with a feedback probe that was connected to a Small Animal Warmer and Thermometer (Bio Research Center Co., Ltd., Aichi, Japan). A dental trephine drill was used to perform a craniotomy of 5 mm in diameter over the left parietal cortex, 6 mm posterior to the coronal suture, and 6 mm lateral to the sagittal suture. A CCI device (Amscien Instruments LLC, Richmond, VA, USA) was used to induce TBI in the mice in the injury group, according to a previously described method [32]. A pneumatic piston with a 3-mm-diameter rounded metal tip was aligned vertically at 15° over the center of the craniotomy so that the tip was perpendicular to the dural surface at impact. The CCI device was set at 20 psi for low pressure in order to hold the piston rod at its highest position, and 100 psi for high pressure to rapidly move the cylindrical rod to its lowest position, resulting in a velocity of 3.54 m/s and a deformation depth of 2 mm below the dura. Immediately after the TBI, the bone flap was adhered to a plastic plate (7-mm diameter, 0.2-mm thickness) and restored in order to seal the craniotomy, and the scalp was sutured closed. The mice were placed in a heat-controlled cage in order to maintain their body temperature while they were recovering from the anesthesia, following i.p. injection of Antisedan (0.3 mg/kg, ZENOAQ) to antagonize the Dormitol [33]. The mice in the sham group underwent a craniotomy but no CCI treatment. The mice in the control group received neither a craniotomy nor CCI treatment [34].

### Sample preparation

The mice were perfused transcardially with 4% paraformaldehyde phosphate buffer solution (PFA, Wako Pure Chemical Industries) while they were under anesthesia using 150 mg/kg pentobarbital (i.p.) and 3% isoflurane (Wako Pure Chemical Industries) delivered using the facemask of an inhalation anesthesia apparatus (Narcobit-E) 1, 4, 7, and 14 days after surgery (for injury, sham, and control groups: *n* = 5 per time point, total = 45). The brains were removed and postfixed in 4% PFA at 4°C overnight, immersed in a 15% sucrose solution for one day, placed in a 30% sucrose solution for another day at 4°C, then cut coronally into 4-mm–thick sections using a mouse brain slicer (Neuroscience Inc., Tokyo, Japan). The coronal brain sections were photographed with a digital camera in order to confirm that the size of the lesion was uniform in each mouse. The coronal brain sections were then processed with optimal cutting temperature (OCT) compound (Tissue-Tek, Sakura Finetek Japan Co., Ltd., Tokyo, Japan) embedding, sectioned (15-μm thickness), and mounted on siliconized slides (Matsunami Glass Ind., Ltd., Tokyo, Japan) with the ipsilateral side on the brain sections on the left and the contralateral side on the same sections that were without damage and used as control tissue on the right.

For total RNA extraction, the mice were transcardially perfused with phosphate-buffered saline (PBS, pH 7.4) while under anesthesia using 150 mg/kg pentobarbital (i.p.) and 3% isoflurane (Wako Pure Chemical Industries) delivered using the facemask of an inhalation anesthesia apparatus (Narcobit-E) 1, 4, 7, and 14 days after surgery (for injury, sham, and control groups: *n* = 5 per time point, total = 45). The brains were removed, cut into coronal blocks (3-mm square) by area, and stored at −80°C until they were used for extraction.

### Histochemical staining

Senescence-associated-β-galactosidase (SA-β-gal) staining was performed on coronal sections containing the penumbral area of the injured cerebrum using a Cellular Senescence Assay Kit (CBA-230; Cell Biolabs Inc., San Diego, CA, USA) according to the manufacturer’s protocol with minor modifications. Brain sections for PFA fixed that were stained for SA-β-gal were photographed with a camera that was attached to a light microscope (FSX100; Olympus Corporation, Tokyo, Japan) to assess the penumbra of the injured cerebrum on the side ipsilateral and contralateral in the injury, sham, and control group. Areas measuring 0.44 mm × 0.32 mm (length × width) from each image were scanned. We selected type 8-bit, used the “adjust,” “threshold max 180”, and “measure” commands in ImageJ (National Institutes of Health, Bethesda, MD, USA). The results of “% area” were used as the stained level of SA-β-gal [31, 32].

### Immunohistochemistry

For single immunohistochemistry-based observation of the penumbral area of the injured cerebrum, we used the primary antibodies against cyclin D1 (ab16663; Abcam, Tokyo, Japan; 1:5000), PCNA (ab92552; Abcam; 1:100,000), CDKN2A (p16, PA5-20379; Thermo Fisher Scientific Inc., Tokyo, Japan; 1:5000), and p21 (ab107099; Abcam; 1:2000). Sections of the brain were incubated in primary antibodies overnight at 4°C and then incubated at room temperature for 30 min with the universal immunoperoxidase polymer for mouse tissue sections for anti each primary antibody (Histofine Simple Stain Mouse MAX PO, Nichirei Biosciences Inc., Tokyo, Japan), followed by enzymatic development using the 3,3′-diaminobenzidine (DAB) Substrate Kit (ACH500, ScyTek Laboratories, Logan, UT, USA) and counterstaining with hematoxylin. To optimize the experimental conditions and reduce variability, all sections were treated similarly, enabling the semiquantitative measurement of the labeling [35]. Brain sections that were stained for each protein were photographed with a camera that was attached to a light microscope (FSX100) to assess the penumbra of the injured cerebrum on the side ipsilateral and contralateral in the injury, sham, and control groups. Areas 0.22 mm × 0.16 mm (length × width) and 0.11 mm × 0.08 mm (length × width) from each image were scanned.

We analyzed sections of the ipsilateral side in the injury group (−1.46 mm from the bregma). All immunopositive cells in the ipsilateral hemicerebrum exhibiting penumbra were photomicrographed, and the number of immunopositive cell bodies was counted by ImageJ. Photomicrographs were obtained using the stitching function of the camera and the light microscope (FSX100) according to the manufacturer’s protocol. The stitching function was carried out on 5 × 5 pictures such that a 1.98 mm × 1.55 mm (length × width) area was scanned as a color image. Using ImageJ to count immunopositive cell bodies, firstly, we separated the nuclei stained by hematoxylin and the immunopositive cell stained by DAB in the color images using the Color Deconvolution plugin. We then used the “adjust,” “threshold,” and “analyze particles” commands in ImageJ for DAB color. The “analyze particles” settings were set to sizes of 28–infinity for the immunopositive cells indicated by the DAB staining. Circularity was set to 0.00–1.00 [31]. We also confirmed the results by manually counting the immunopositive cells on photomicrographs.

Double immunohistochemistry-based analyses of the penumbral area of the injured cerebrum were performed to confirm whether proteins of cyclin D1, PCNA, p16, p21 co-localized with neurons, astrocytes, and microglia. The primary antibodies also included antibodies against glial fibrillary acidic protein (GFAP; 345860; Millipore, Tokyo, Japan; 1:4000), ionized calcium binding adaptor molecule 1 (Iba 1; ab107159; Abcam; 1:5000), and NeuN (ABN90; MerckMillipore, Tokyo, Japan; 1:6000). Sections of the brain were incubated in primary antibodies overnight at 4°C, and then incubated at room temperature for 60 min with antibodies against each primary antibody labeled with Alexa flour 488 (goat anti-rabbit A11034, donkey anti-rabbit IgG A21206, donkey anti-Rat A21208, IgG secondary antibody, Thermo Fisher Scientific Inc.; 1:2000) and with Alexa flour 568 (goat anti-rat A11077, donkey anti-goat A11057, goat anti-guinea pig A11075, donkey anti-rabbit A10042, IgG secondary antibody, Thermo Fisher Scientific Inc.; 1:2000), then mounted using Dapi-Fluoromont-G (SouthernBiotech, Birmingham, AL, USA). To optimize the experimental conditions and reduce variability, all sections were treated similarly, enabling the semiquantitative measurement of the labeling [35]. Brain sections were photographed using a scanner that was attached to a confocal scanning microscope (LSM-710; ZEISS Corporation, Oberkochen, Germany). Areas 0.09 mm × 0.09 mm (length × width) from each image were scanned.

### Real-time PCR

A 30-mg sample was taken from brain tissue cut from the area adjacent to the cerebrum contusion in the injury group and a corresponding area of the brain in the sham and control groups. Total RNA was extracted from the samples using an AllPrep DNA/RNA/Protein Mini Kit (Qiagen, Tokyo, Japan), according to the manufacturer’s protocol. The purity of the total RNA was determined using an ultraviolet spectrophotometer at wavelengths of 260 and 280 nm (NanoDrop 2000, Thermo Fisher Scientific Inc.). The RNA quality was verified on a 3-(N-morpholino)-propanesulfonic acid (MOPS)-formaldehyde-agarose gel. The cDNA for real-time PCR was created using a High Capacity cDNA Reverse Transcription Kit (Thermo Fisher Scientific Inc.) according to the manufacturer’s protocol. Real-time PCR was performed on a StepOnePlus Real-Time PCR System (Thermo Fisher Scientific Inc.) with the PowerUp SYBR Green PCR Master Mix (Thermo Fisher Scientific Inc.). The sequences for the primers were constructed using the Primer-Basic Local Alignment Search Tool (BLAST; National Center for Biotechnology Information, Bethesda, MD, USA) as shown in Table 1. The cycling parameters were as follows: thermal activation for 2 min at 50°C and 95°C, and 50 cycles of PCR (denaturing for 15 sec at 95°C, annealing 15 sec at 60°C, and extension for 1 min at 72°C). Multi housekeeping genes were used as internal standard. The ratio of mRNA expression of each target transcription to those of glyceraldehyde-3-phosphate dehydrogenase (*Gapdh*), peptidylprolyl isomerase A (*Ppia*), and hypoxanthine guanine phosphoribosyl transferase (*Hprt*) was compared among the groups [35,36].

**Table 1:** Sequences of the primers for real time PCR.

Transcript name	Primer	Sequence	Access number
Cyclin D1	Forward	TCAAGTGTGACCCGGACTG	NM_007631.2
	Reverse	CTCCAGAAGGGCTTCAATCTGT	
PCNA	Forward	ATCTAGTCGCCACAACTCCG	NM_011045.2
	Reverse	CGTGAGACGAGTCCATGCTC	
p16	Forward	GTGTGCATGACGTGCGG	NM_001040654.1
	Reverse	CACCTGAATCGGGGTACGAC	
p21	Forward	GTGAGGAGGAGCATGAATGGA	NM_001111099.2
	Reverse	GCACCTTTTATTCTGCTGGCAA	
p53	Forward	TCATCCCTCCCCTTTTCTGTC	NM_001127233.1
	Reverse	ATGGCGGGAAGTAGACTGGC	
PPIA	Forward	TTTCCGACTGTGGACAGCTCTA	NM_008907.1
	Reverse	AATGCCCGCAAGTCAAAAGA	
HPRT	Forward	AAGCCTAAGATGAGCGCAAGT	NM_013556.2
	Reverse	ACAGGACTCCTCGTATTTGCAG	
GAPDH	Forward	AAATGAGAGAGGCCCAGCTACTC	NM_008084.3
	Reverse	GGAGGGCTGCAGTCCGTAT	

*Pcna*: proliferating cell nuclear antigen. p16: cyclin-dependent kinase inhibitor 2A. p21: cyclin-dependent kinase inhibitor 1A. p53: transformation related protein 53. *Ppia*: peptidylprolyl isomerase A. *Hprt*: hypoxanthine guanine phosphoribosyl transferase. *Gapdh*: glyceraldehyde-3-phosphate dehydrogenase.

### Statistical analysis

All values are reported as the means ± standard deviation. A two-way analysis of variance was used to assess the differences between the ipsilateral and contralateral sides of the brain sections or the mRNA expression of the ipsilateral sides of the brain between the injury, sham, and control groups over the different periods. Alternately, a one-way analysis of variance was used to assess the differences in the ipsilateral sides of the brain sections in the injury group between the different periods, and, if significant, this was followed with a Bonferroni post-hoc test. Differences were considered significant with *p* values less than 0.05.

## Results

### SA-β-galactosidase

**Fig 1:**
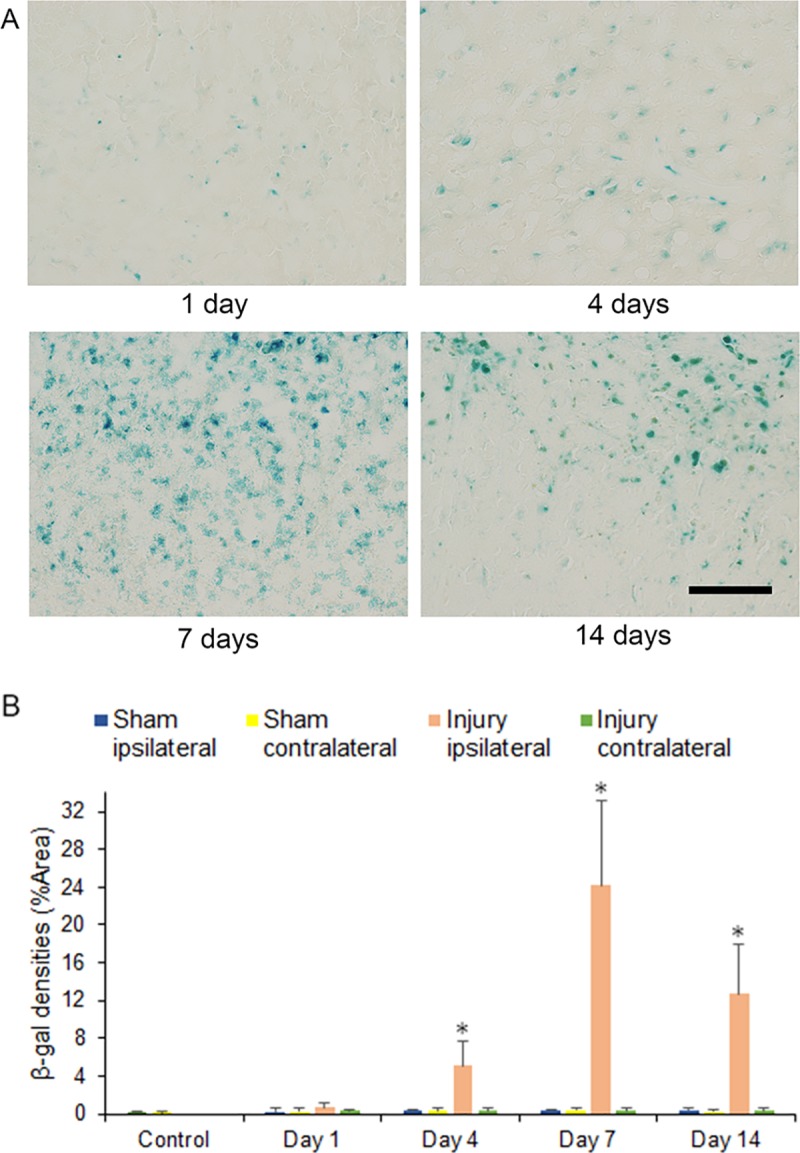
Senescence-associated-β-galactosidase (SA-β-gal) staining in the cerebrum 1 to 14 days after injury. (A) SA-β-gal-positive cells could be observed in the ipsilateral cerebrum at 1, 4, 7, and 14 days after injury in injury groups. Scale = 100 μm. (B) Stained levels of SA-β-gal-positive cells, digitized for analysis by ImageJ software, in the ipsilateral side of the brain injury in the injury group at 4, 7, and 14 days after injury as compared to that in the corresponding area of the contralateral hemicerebrum of the injury group and the bilateral hemicerebrum of the sham and control groups (*p < 0.05, *n* = 5 for control group, *n* = 5 for the sham group, n = 5 for the injury group).

SA-β-gal-positive cells were noted in the area adjacent to the hemicerebrum contusion including the hippocampus during the first 14 days. The levels of SA-β-gal-positive cell staining within the ipsilateral side of the brain injury in the injury group gradually increased from 1 to 14 days and peaked at 7 days after injury. These levels were significantly increased at 4, 7, and 14 days after injury compared to that in the corresponding area of the contralateral hemicerebrum of the injury group and the bilateral hemicerebrum of the sham and control groups at 1 to 14 days after injury (14.25-, 62.76-, and 36.78-fold increase at 4, 7, and 14 days, respectively; *p* < 0.05) (Fig 1).

### Immunohistochemistry

**Fig 2:**
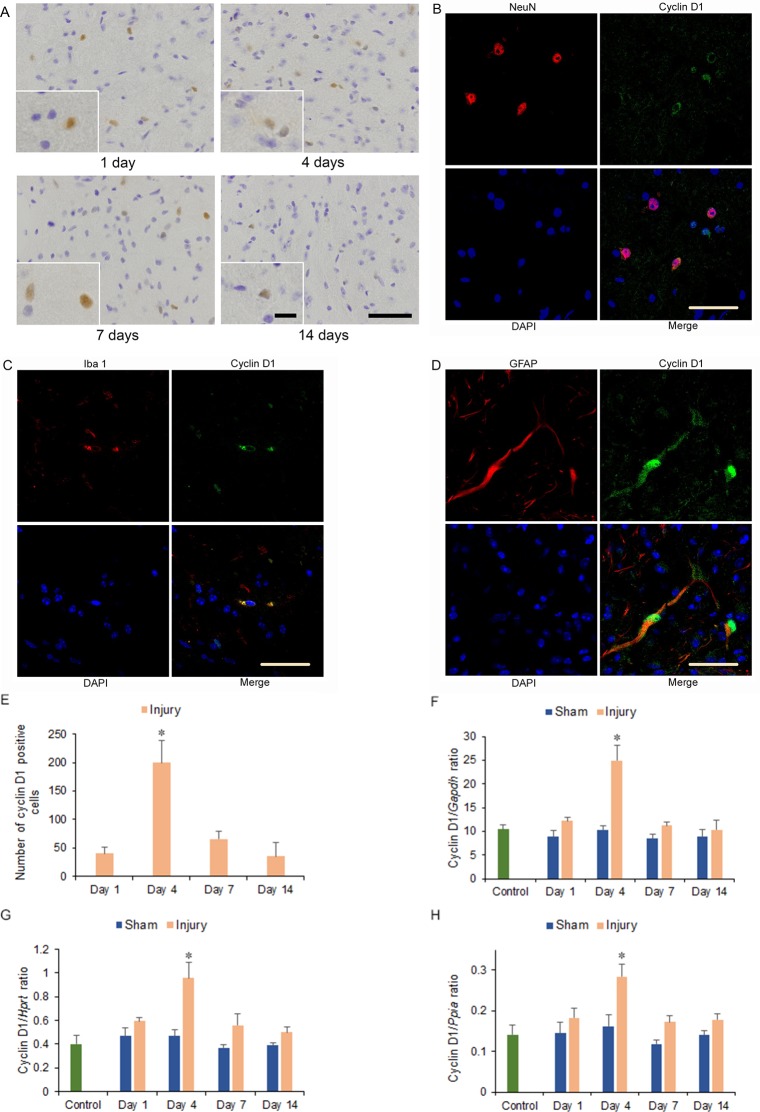
Cyclin D1 immunohistochemistry and mRNA expression in the cerebrum 1 to 14 days after injury. (A) Cyclin D1 immunostained cells could be observed in the ipsilateral hemicerebrum at 1, 4, 7, and 14 days after injury in injury groups. Long scale = 50 μm, short scale = 12.5 μm. (B-D) Double immunohistochemistry of the ipsilateral hemicerebrum at 4 days after injury for co-localization of (B) cyclin D1, NeuN, DAPI (4',6-diamidino-2-phenylindole), and merge; (C) cyclin D1, ionized calcium binding adaptor molecule 1 (Iba 1), DAPI, and merge; and (D) cyclin D1, glial fibrillary acidic protein (GFAP), DAPI, and merge. Scale = 30 μm. (E) Graph of the numbers of cyclin D1 immunostained cells from (A). (F-H) Cyclin D1 mRNA expression in the ipsilateral hemicerebrum 1 to 14 days after injury. Expressions were normalized to that of glyceraldehyde-3-phosphate dehydrogenase (*Gapdh*) (F), hypoxanthine guanine phosphoribosyl transferase (*Hprt*) (G), and peptidylprolyl isomerase A (*Ppia*) (H) (*p < 0.05, *n* = 5 for control group, *n* = 5 for the sham group, *n* = 5 for the injury group).

**Fig 3:**
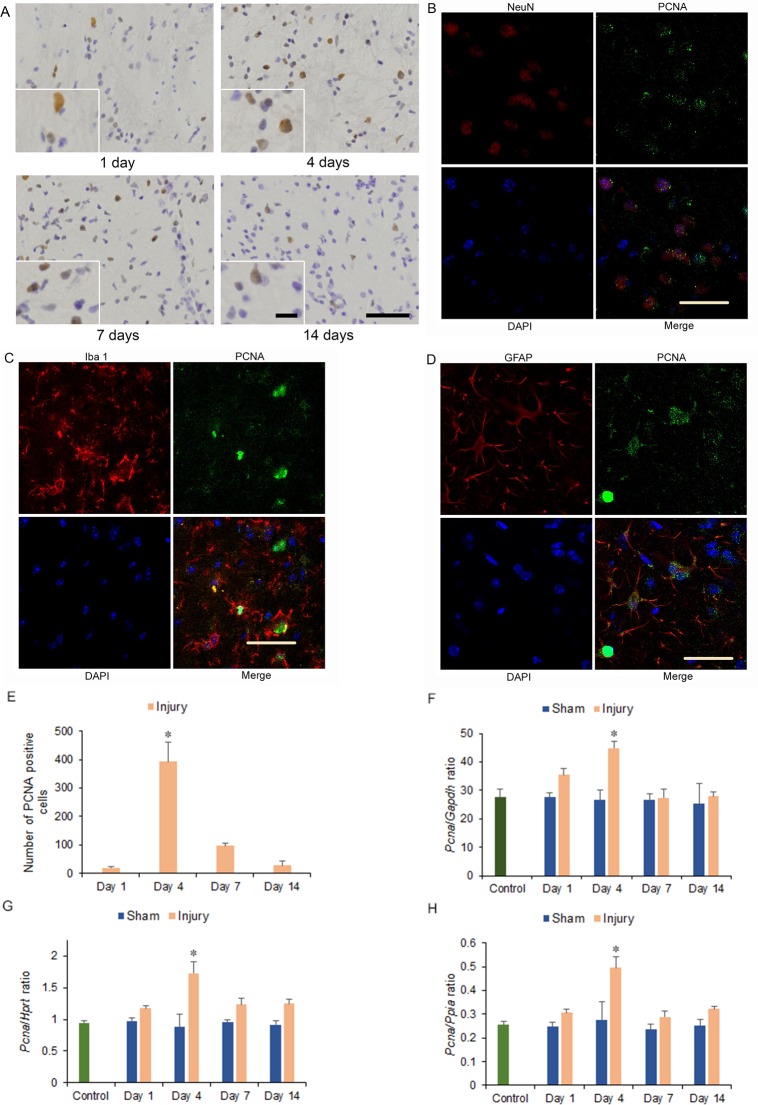
Proliferating cell nuclear antigen (PCNA) immunohistochemistry and mRNA expression in the cerebrum 1 to 14 days after injury. (A) PCNA immunostained cells could be observed in the ipsilateral hemicerebrum at 1, 4, 7, and 14 days after injury in injury groups. Long scale = 50 μm, short scale = 12.5 μm. (B-D) Double immunohistochemistry of the ipsilateral hemicerebrum at 4 days after injury for co-localization of (B) PCNA, NeuN, DAPI (4',6-diamidino-2-phenylindole), and merge; (C) PCNA, ionized calcium binding adaptor molecule 1 (Iba 1), DAPI, and merge; and (D) PCNA, glial fibrillary acidic protein (GFAP), DAPI, and merge. Scale = 30 μm. (E) Graph of the numbers of PCNA immunostained cells in (A). (F-H) *Pcna* mRNA expression in the ipsilateral hemicerebrum 1 to 14 days after injury. *Pcna* mRNA expressions were normalized to that of glyceraldehyde-3-phosphate dehydrogenase (*Gapdh*) (F), hypoxanthine guanine phosphoribosyl transferase (*Hprt*) (G), and peptidylprolyl isomerase A (*Ppia*) (H) (*p < 0.05, *n* = 5 for control group, *n* = 5 for the sham group, *n* = 5 for the injury group).

Cyclin D1 and PCNA immunostained cells were observed among neurons, astrocytes, and microglia of the hemicerebrum on the ipsilateral side of the brain injury in the injury group at 1 to 14 days after injury. Moreover, more cyclin D1 and PCNA immunostained cells were observed at 4 and 7 days than at 1 and 14 days after injury, and at 4 compared to 7 days after injury, with significantly increased numbers at 4 days after injury compared with the other days (*p* < 0.05) (Figs 2A, 2E, 3A and 3E). However, cyclin D1 and PCNA immunostained cells were not observed in the area adjacent to the hemicerebrum contusion in the corresponding area of the contralateral hemicerebrum of the injury group as same as the bilateral hemicerebrum of the sham and control groups at 1 to 14 days after injury (S1 Fig, S2 Fig).

**Fig 4:**
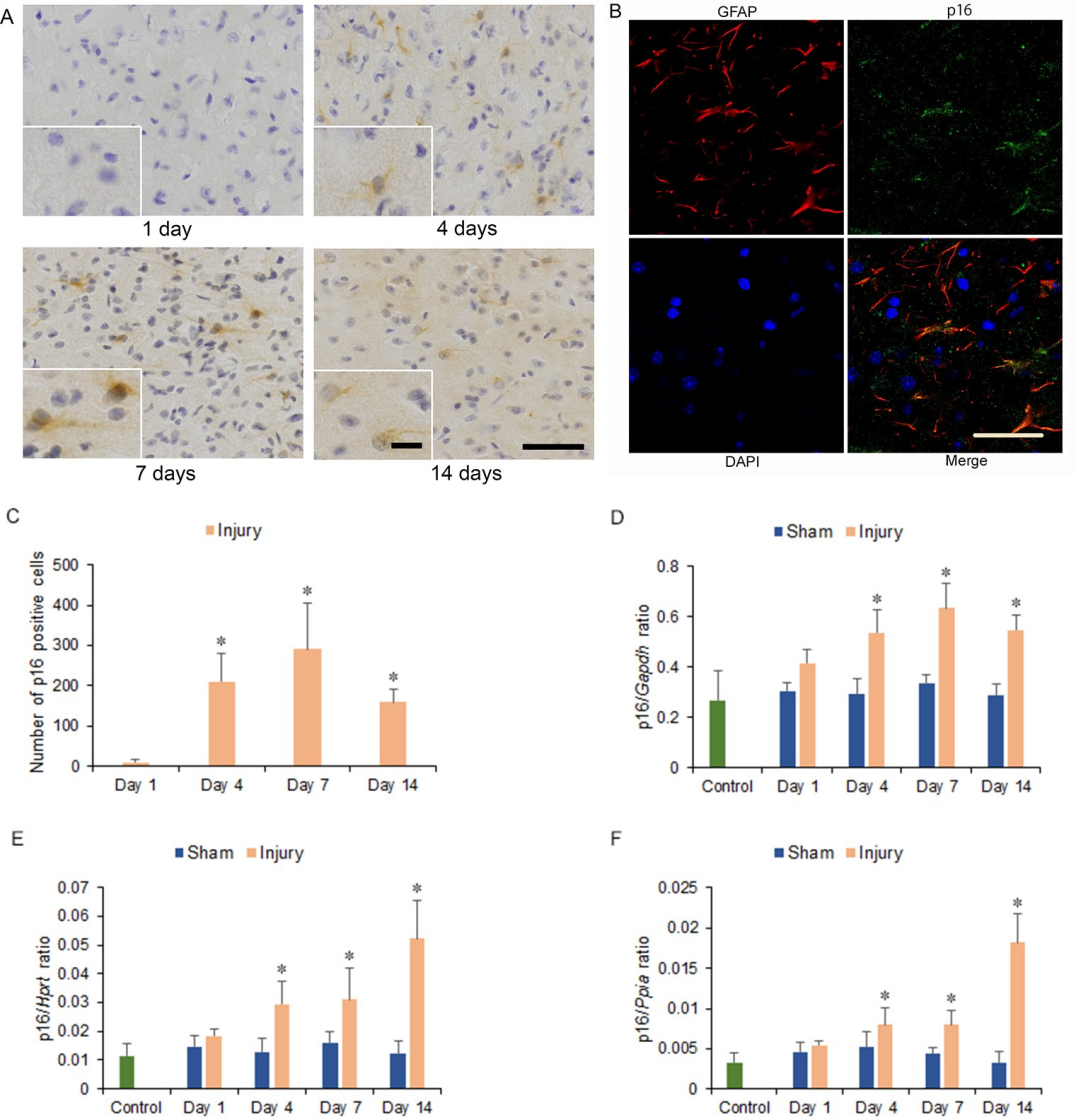
Cyclin-dependent kinase inhibitor 2A (p16) immunohistochemistry and mRNA expression in the cerebrum 1 to 14 days after injury. (A) p16 immunostained cells could be observed in the ipsilateral hemicerebrum at 4, 7, and 14 days after injury in injury groups. Long scale = 50 μm, short scale = 12.5 μm. (B) Double immunohistochemistry of the ipsilateral hemicerebrum at 7 days after injury for co-localization of p16, glial fibrillary acidic protein (GFAP), DAPI (4',6-diamidino-2-phenylindole), and merge. Scale = 30 μm. (C) Graph of the numbers of p16 immunostained cells in the ipsilateral hemicerebrum in (A). (D-F) p16 mRNA expression in the ipsilateral hemicerebrum 1 to 14 days after injury. p16 mRNA expressions were normalized to that of glyceraldehyde-3-phosphate dehydrogenase (*Gapdh*) (D), hypoxanthine guanine phosphoribosyl transferase (*Hprt*) (E), and peptidylprolyl isomerase A (*Ppia*) (F) (*p < 0.05, *n* = 5 for control group, *n* = 5 for the sham group, *n* = 5 for the injury group).

The p16 immunostained cells were observed in astrocytes of the hemicerebrum on the ipsilateral side of the brain injury in the injury group at 1, 4, 7, and 14 days after injury (Fig 4A and 4B), without observing in neuron and microglia (S3A and S3B Fig). Moreover, cells were most frequently observed at 7 days after injury. The numbers of p16 immunostained cells significantly increased at 4, 7, and 14 days compared with that at 1 day after injury (*p* < 0.05) (Fig 4C). However, p16 immunostained cells were not observed in the area adjacent to the hemicerebrum contusion in the corresponding area of the contralateral hemicerebrum of the injury group as same as the bilateral hemicerebrum of the sham and control groups at 1 to 14 days after injury (S3C Fig).

**Fig 5:**
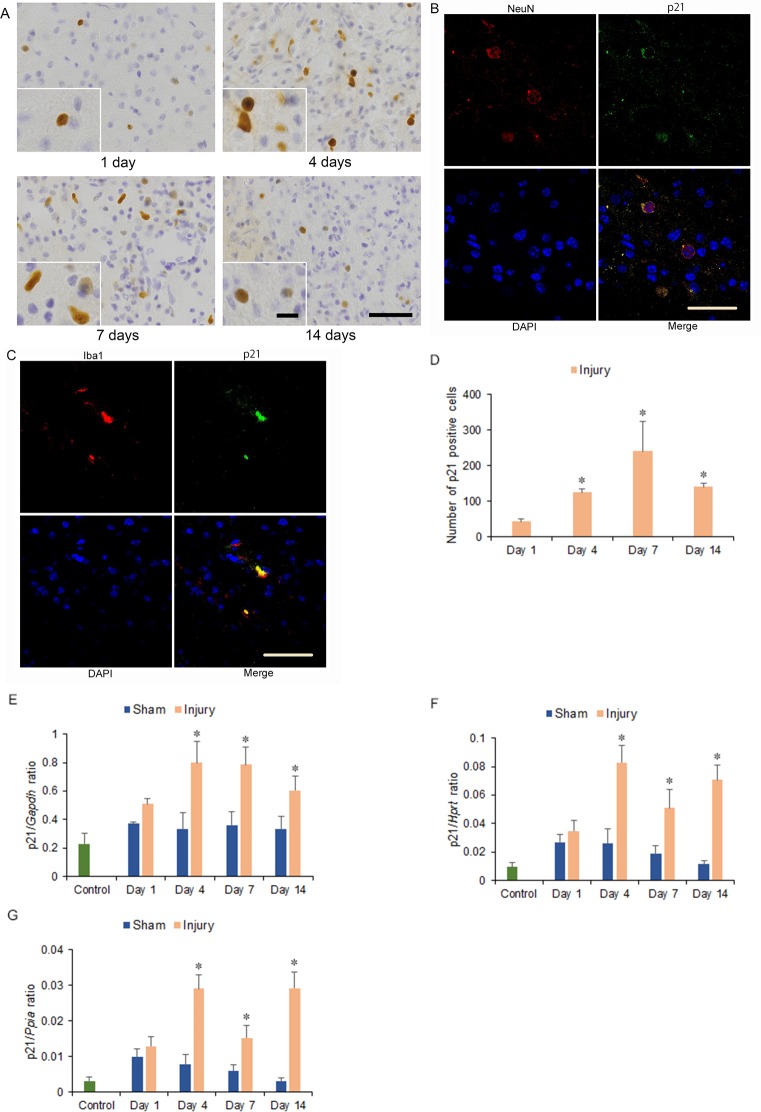
Cyclin-dependent kinase inhibitor 1A (p21) immunohistochemistry and mRNA expression in the cerebrum 1 to 14 days after injury. (A) p21 immunostained cells could be observed in the ipsilateral hemicerebrum at 1, 4, 7, and 14 days after injury in injury groups. Long scale = 50 μm, short scale = 12.5 μm. (B, C) Double immunohistochemistry of the ipsilateral hemicerebrum at 7 days after injury for co-localization of (B) p21, NeuN, DAPI (4',6-diamidino-2-phenylindole), and merge; and (C) p21, ionized calcium binding adaptor molecule 1 (Iba 1), DAPI, and merge. Scale = 30 μm. (D) Graph of the numbers of p21 immunostained cells in the ipsilateral hemicerebrum in the injury group at 1 to 14 days after injury in (A). (E-G) p21 mRNA expression in the ipsilateral hemicerebrum 1 to 14 days after injury. p21 mRNA expressions were normalized to that of glyceraldehyde-3-phosphate dehydrogenase (*Gapdh*) (E), hypoxanthine guanine phosphoribosyl transferase (*Hprt*) (F), and peptidylprolyl isomerase A (*Ppia*) (G) (*p < 0.05, *n* = 5 for control group, *n* = 5 for the sham group, *n* = 5 for the injury group).

The p21 immunostained cells were observed in the neurons and microglia of the hemicerebrum on the ipsilateral side of the brain injury in the injury group at 1 to 14 days after injury (Fig 5A–5C), without observing in astrocyte (S4A Fig). Moreover, significantly more p21 immunostained cells were observed at 4, 7, and 14 days than at 1 day after injury (*p* < 0.05) (Fig 5D). However, p21 immunostained cells were not observed in the area adjacent to the hemicerebrum contusion in the corresponding area of the contralateral hemicerebrum of the injury group as same as the bilateral hemicerebrum of the sham and control groups at 1 to 14 days after injury (S4B Fig).

### mRNA expression

Cyclin D1 mRNA expression increased significantly on the ipsilateral side of the cerebrum in the injury group at 4 days after injury compared with that in the injury group at the other days and the sham and control groups at 1 to 14 days after injury (2.67-fold increase for cyclin D1/*Gapdh* ratio, 2.28-fold increase for cyclin D1/*Hprt* ratio, and 2.00-fold increase for cyclin D1/*Ppia* ratio, respectively; *p* < 0.05) (Fig 2F–2H).

*Pcna* mRNA expression increased significantly within the ipsilateral side of the cerebrum in the injury group at 4 days after injury compared with that in the injury group at other days and the sham and control groups at 1 to 14 days after injury (1.60-fold increase for *Pcna*/*Gapdh* ratio, 1.85-fold increase for *Pcna*/*Hprt* ratio, and 1.99-fold increase for *Pcna*/*Ppia* ratio, respectively; *p* < 0.05) (Fig 3F–3H).

**Fig 6:**
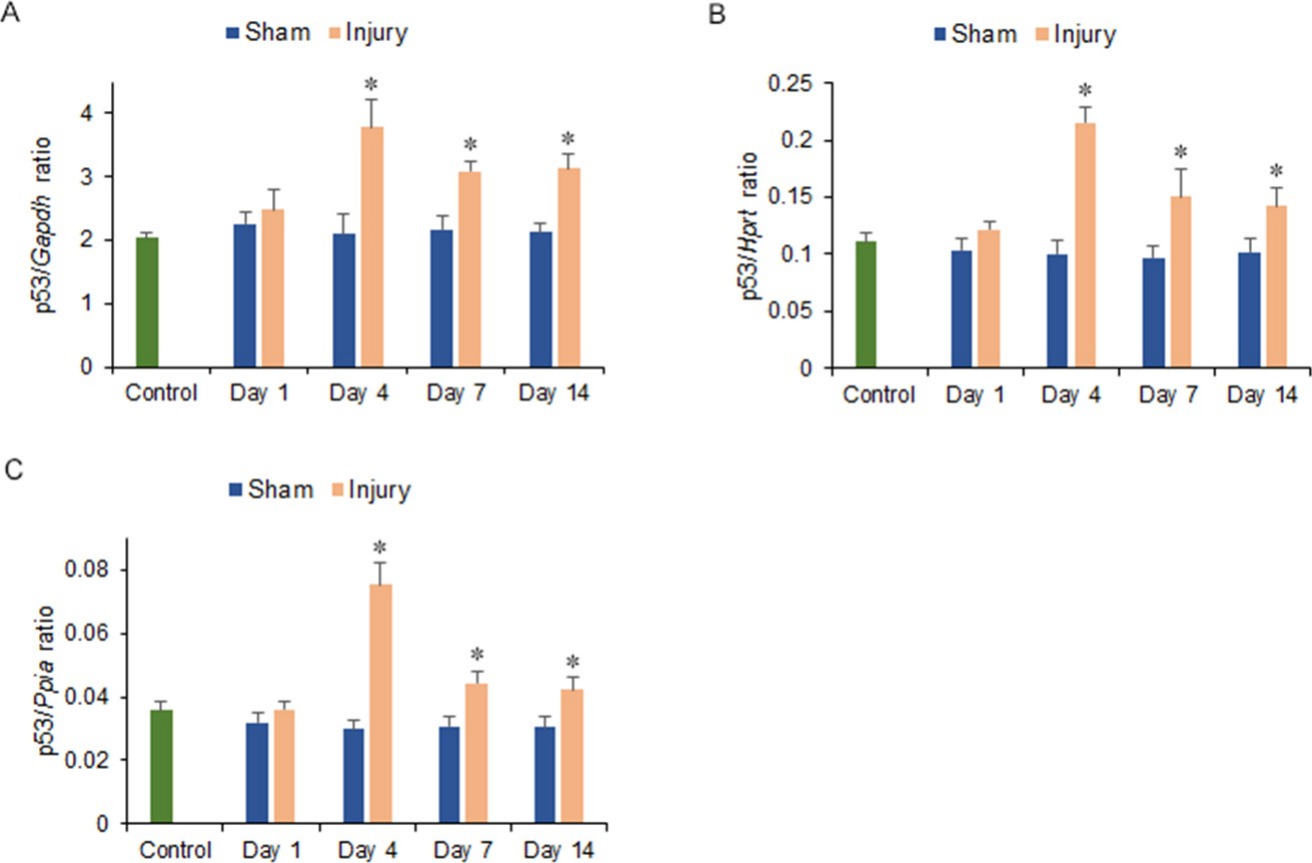
mRNA expression of the transformation related protein 53 (p53) in the ipsilateral hemicerebrum 1 to 14 days after injury. p53 mRNA expressions were normalized to that of glyceraldehyde-3-phosphate dehydrogenase (*Gapdh*) (A), hypoxanthine guanine phosphoribosyl transferase (*Hprt*) (B) and peptidylprolyl isomerase A (*Ppia*) (C) (*p < 0.05, *n* = 5 for control group, *n* = 5 for the sham group, *n* = 5 for the injury group).

The levels of p16, p21, and p53 mRNA expression increased in the injury group from 4 to 14 days after injury. Significant increases were observed within the ipsilateral side of the cerebrum in the injury group at 4, 7, and 14 days after injury compared with the injury group at 1 day and the sham and control groups at 1 to 14 days after injury for p16 (1.80-, 2.13-, and 1.84-fold increase for p16/*Gapdh* ratio; 2.18-, 2.30-, and 3.87-fold increase for p16/*Hprt* ratio; 1.94-, 1.92-, and 4.39-fold increase for p16/*Ppia* ratio; at 4, 7, and 14 days, respectively. *p* < 0.05) (Fig 4D–4F); for p21 (2.44-, 2.41-, and 1.85-fold increase for p21/*Gapdh* ratio; 4.45-, 2.74-, and 3.81-fold increase for p21/*Hprt* ratio; 4.80-, 2.52-, and 4.82-fold increase for p21/*Ppia* ratio; at 4, 7, and 14 days, respectively; *p* < 0.05) (Fig 5E–5G) and for p53 (1.77-, 1.44-, and 1.46-fold increase for p53/*Gapdh* ratio; 2.10-, 1.47-, and 1.39-fold increase for p53/*Hprt* ratio; 2.38-, 1.40-, and 1.33-fold increase for p53/*Ppia* ratio; at 4, 7, and 14 days, respectively; *p* < 0.05) (Fig 6A–6C).

## Discussion

In this study, the levels of SA-β-gal-positive cell staining within the area adjacent to the cerebrum contusion increased significantly at 4, 7, and 14 days after injury. The numbers of cyclin D1 and PCNA immunostained cells, which included neurons, astrocytes, and microglia of the hemicerebrum on the ipsilateral side of the brain injury in the injury group, increased significantly at 4 days after injury and correlated with significant increases in cyclin D1 and *Pcna* mRNA expression within the area adjacent to the cerebrum contusion. The numbers of p16 immunostained cells, which were observed in astrocytes of the hemicerebrum on the ipsilateral side of the brain injury in the injury group, increased significantly at 4, 7, and 14 days after injury and correlated with significantly increased p16 mRNA expression within the area adjacent to the cerebrum contusion. The numbers of p21 immunostained cells, including neurons and microglia of the hemicerebrum on the ipsilateral side of the brain injury in the injury group, increased significantly at 4, 7, and 14 days after injury and correlated with significantly increased p21 mRNA expression within the area adjacent to the cerebrum contusion. Furthermore, mRNA expression within the area adjacent to the cerebrum contusion increased significantly for p53 at 4, 7, and 14 days.

Timaru-Kast at al. reported that there are housekeeping genes that stably express with respect to age and TBI, PPIA and HPRT are the best combinations of genes in young and old or only young animals, PPIA and GAPDH is the most suitable combination of genes in old mice [36]. In the present study, we used multi housekeeping genes of GAPDH, HPRT, and PPIA as an internal standard. The time point of the peak of the ratio of expression level of the target genes depending on the housekeeping genes were a few variations, but an expression of the target genes significantly increased and correlated at the same time point. Our data supported results by Timaru-Kast at al. and were consistent with the stable expression of GAPDH, HPRT, and PPIA at age and TBI.

TBI increases the expression of cell cycle-related proteins, and the activated cell cycle induces proliferation of astrocytes and microglia to release inflammatory factors [6]. Similar to previous studies in CCI model mice [4], in this study, the expression of cyclin D1 and PCNA mRNA and protein within the area adjacent to the hemicerebrum contusion also increased significantly at 4 days after injury, confirming initial TBI-mediated cell cycle activation until 4 days post-injury. Subsequently, the levels of stained SA-β-gal-positive cells within the area adjacent to the hemicerebrum contusion including the hippocampus increased significantly at 4, 7, and 14 days after injury. Our data were consistent with previous findings that β-gal was observed in many cell types in the ipsilateral hippocampus in a mouse CCI model [27]. As SA-β-gal activity is a senescence marker, the findings of the present study thus suggest that cells with an activated cell cycle may proceed to cellular senescence within the area adjacent to the hemicerebrum contusion after TBI [24, 25].

Two senescence pathways have been identified, namely, the p16-RB and p53-mediated pathways. In particular, p16 is upregulated to inhibit CDK4/6, which maintains RB activation to downregulate E2F, a transcription factor that regulates the cell cycle, thereby arresting cell cycle progression. Alternately, senescence signaling activates p53 to upregulate p21, which inhibits CDK2, thereby downregulating cell proliferation [18, 37].

It has been reported that the expression of p16 protein in astrocytes of the injured cortex increases after TBI as shown by immunohistochemistry in a rat needle injury model [38]. Consistent with this, in the present study we found that p16 immunostained cells were observed in astrocytes of the hemicerebrum on the ipsilateral side of the brain injury in the injury group and that p16 protein and mRNA expression within the area adjacent to the hemicerebrum contusion increased significantly at 4, 7, and 14 days after injury. This result suggests that after 4 days post-injury, astrocytes may undergo senescence via the p16 pathway.

In comparison, in a rat CCI model study, p53 mRNA in the hippocampus ipsilateral to injury increased at 3, 7, and 14 days after injury [29]. Expression of p21 mRNA in injured cortex and hippocampus also increased after TBI by *in situ* hybridization in a rat weight drop model study [28]. Similarly, in the present study, the expression of p53 mRNA and p21 protein and mRNA within the area adjacent to the hemicerebrum contusion increased significantly at 4, 7, and 14 days after injury. In addition, p21 immunostained cells were observed within neurons and microglia of the hemicerebrum on the ipsilateral side of the brain injury in the injury group, which suggested that neurons and microglia may undergo senescence via the p53-p21 pathway from 4 days post-injury.

## Conclusion

Within the area adjacent to the hemicerebrum contusion after TBI, the increase in protein and mRNA expression of cyclin D1 and PCNA up to 4 days after injury suggested that TBI initially activates the cell cycle in neurons, astrocytes, and microglia. Subsequently, the increased SA-β-gal-positive staining score 4 days after TBI suggested that cells with an activated cell cycle may undergo cellular senescence. In particular, astrocytes seemed to have undergone senescence via the p16 pathway 4 days after TBI, whereas the p53-p21 pathway might be active in neurons and microglia.

## Supporting information

S1 Fig Cyclin D1 immunohistochemistry in the cerebrum 1 to 14 days after injury. Cyclin D1 immunostained cells could not be observed in the contralateral hemicerebrum at 1, 4, 7, and 14 days after injury in injury groups. Scale = 50 μm (*n* = 5).
(TIF)

S2 Fig Proliferating cell nuclear antigen (PCNA) immunohistochemistry in the cerebrum 1 to 14 days after injury. PCNA immunostained cells could not be observed in the contralateral hemicerebrum at 1, 4, 7, and 14 days after injury in injury groups. Scale = 50 μm (*n* = 5).
(TIF)

S3 Fig Cyclin-dependent kinase inhibitor 2A (p16) immunohistochemistry in the cerebrum 1 to 14 days after injury. (A-B) Double immunohistochemistry of the contralateral hemicerebrum at 7 days after injury for localization of (A) p16, NeuN, DAPI (4',6-diamidino-2-phenylindole), and merge; and (B) p16, ionized calcium binding adaptor molecule 1 (Iba 1), DAPI, and merge. Scale = 30 μm. (C) p16 immunostained cells could not be observed in the contralateral hemicerebrum at 1, 4, 7, and 14 days after injury in injury groups. Scale = 50 μm (*n* = 5).
(TIF)

S4 Fig Cyclin-dependent kinase inhibitor 1A (p21) immunohistochemistry in the cerebrum 1 to 14 days after injury. (A) Double immunohistochemistry of the contralateral hemicerebrum at 7 days after injury for localization of p21, glial fibrillary acidic protein (GFAP), DAPI (4',6-diamidino-2-phenylindole), and merge. Scale = 30 μm. (B) p21 immunostained cells could not be observed in the contralateral hemicerebrum at 1, 4, 7, and 14 days after injury in injury groups. Scale = 50 μm (*n* = 5).
(TIF)
