# Supplementary figures and images for: Senescence-associated-β-galactosidase staining following traumatic brain injury in the mouse cerebrum
Source: PLoS One. 2019 Mar 11;14(3):e0213673. doi: 10.1371/journal.pone.0213673 (PMC6411151; doi:10.1371/journal.pone.0213673)

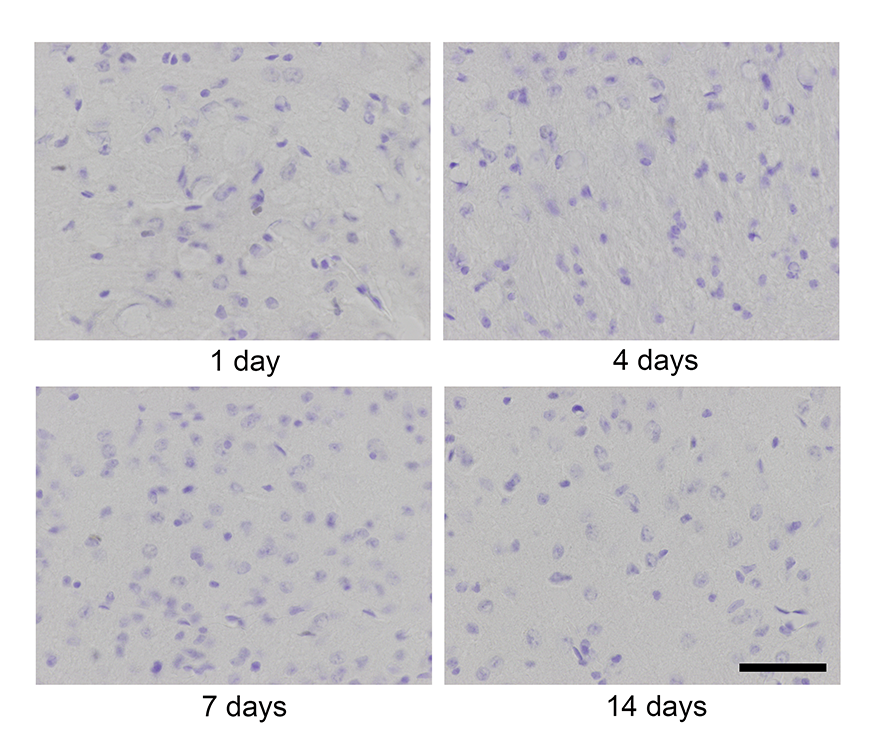

Supplement: S1 Fig — Cyclin D1 immunostained cells could not be observed in the contralateral hemicerebrum at 1, 4, 7, and 14 days after injury in injury groups. Scale = 50 μm (n = 5). (TIF) [file pone.0213673.s001.tif]

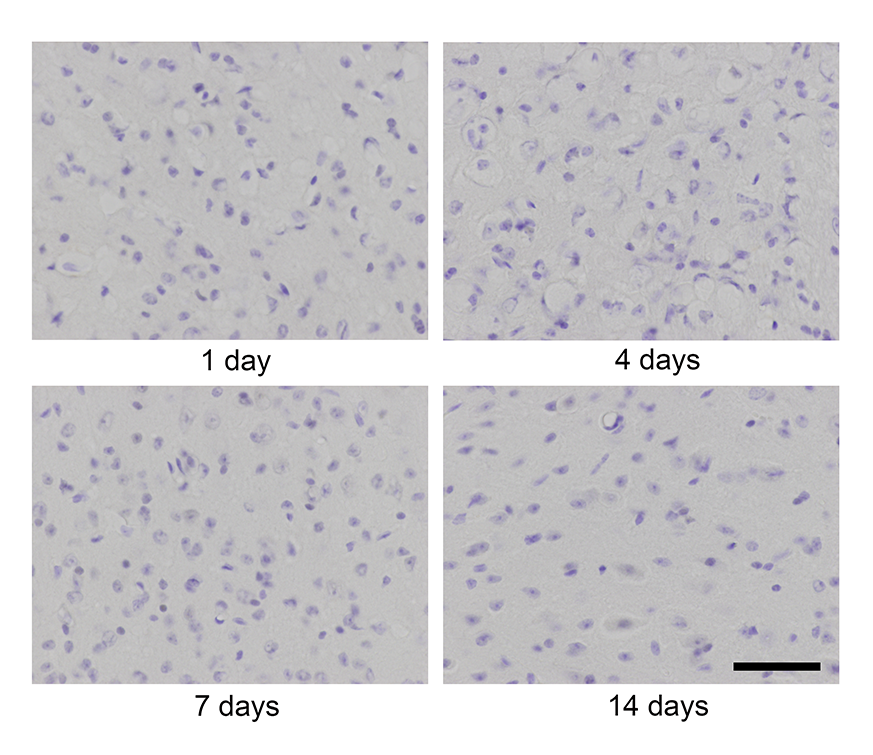

Supplement: S2 Fig — PCNA immunostained cells could not be observed in the contralateral hemicerebrum at 1, 4, 7, and 14 days after injury in injury groups. Scale = 50 μm (n = 5). (TIF) [file pone.0213673.s002.tif]

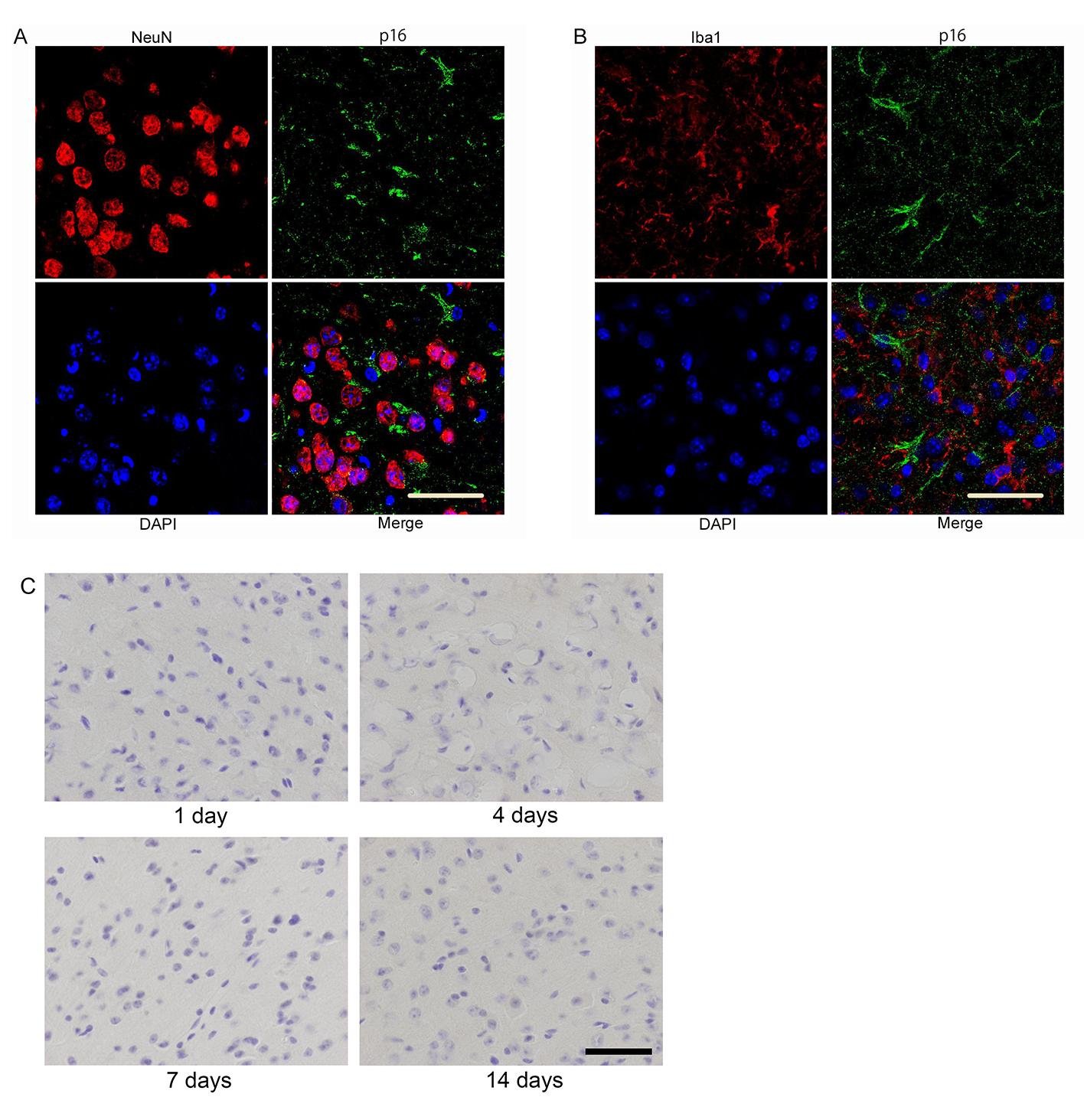

Supplement: S3 Fig — (A-B) Double immunohistochemistry of the contralateral hemicerebrum at 7 days after injury for localization of (A) p16, NeuN, DAPI (4',6-diamidino-2-phenylindole), and merge; and (B) p16, ionized calcium binding adaptor molecule 1 (Iba 1), DAPI, and merge. Scale = 30 μm. (C) p16 immunostained cells could not be observed in the contralateral hemicerebrum at 1, 4, 7, and 14 days after injury in injury groups. Scale = 50 μm (n = 5). (TIF) [file pone.0213673.s003.tif]

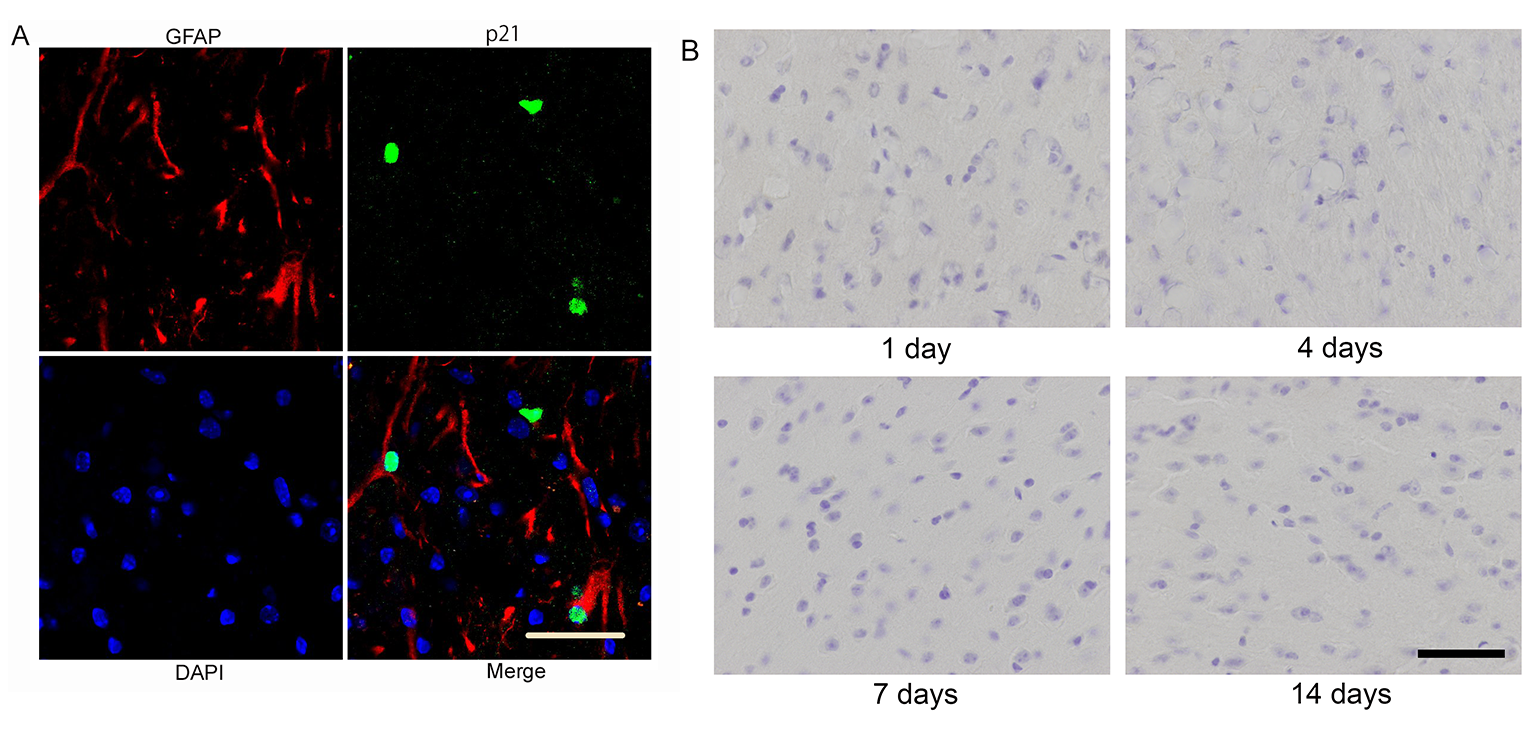

Supplement: S4 Fig — (A) Double immunohistochemistry of the contralateral hemicerebrum at 7 days after injury for localization of p21, glial fibrillary acidic protein (GFAP), DAPI (4',6-diamidino-2-phenylindole), and merge. Scale = 30 μm. (B) p21 immunostained cells could not be observed in the contralateral hemicerebrum at 1, 4, 7, and 14 days after injury in injury groups. Scale = 50 μm (n = 5). (TIF) [file pone.0213673.s004.tif]
